# Serological responses to immunization during nephrosis in infants with congenital nephrotic syndrome of the Finnish type

**DOI:** 10.3389/fped.2024.1392873

**Published:** 2024-05-02

**Authors:** Okko Savonius, Anu Kaskinen, Tuula Hölttä, Elisa Ylinen, Juuso Tainio, Tea Nieminen, Timo Jahnukainen

**Affiliations:** ^1^ Department of Pediatric Nephrology and Transplantation, New Children’s Hospital, Pediatric Research Center, Helsinki University Hospital and University of Helsinki, Helsinki, Finland; ^2^ Department of Pediatric Infectious Diseases, New Children’s Hospital, Pediatric Research Center, Helsinki University Hospital and University of Helsinki, Helsinki, Finland

**Keywords:** congenital nephrotic syndrome, nephrosis, vaccine, immunization, vaccine response, children

## Abstract

**Background:**

Pretransplant vaccination is generally recommended to solid organ transplant recipients. In infants with congenital nephrotic syndrome (CNS), the immune response is hypothetically inferior to other patients due to young age and urinary loss of immunoglobulins, but data on the immunization response in severely nephrotic children remain scarce. If effective, however, early immunization of infants with CNS would clinically be advantageous.

**Methods:**

We investigated serological vaccine responses in seven children with CNS who were immunized during nephrosis. Antibody responses to measles-mumps-rubella -vaccine (MMR), a pentavalent DTaP-IPV-Hib -vaccine (diphtheria, tetanus, acellular pertussis, inactivated poliovirus, *Haemophilus influenzae* type b), varicella vaccine, combined hepatitis A and B vaccine, and pneumococcal conjugate vaccine (PCV) were measured after nephrectomy either before or after kidney transplantation.

**Results:**

Immunizations were started at a median age of 7 months [interquartile range (IQR) 7–8], with a concurrent median proteinuria of 36,500 mg/L (IQR 30,900–64,250). Bilateral nephrectomy was performed at a median age of 20 months (IQR 14–25), and kidney transplantation 10–88 days after the nephrectomy. Antibody levels were measured at median 18 months (IQR 6–23) after immunization. Protective antibody levels were detected in all examined children for hepatitis B (5/5), *Clostridium tetani* (7/7), rubella virus (2/2), and mumps virus (1/1); in 5/6 children for varicella; in 4/6 for poliovirus and vaccine-type pneumococcal serotypes; in 4/7 for *Haemophilus influenzae* type B and *Corynebacterium diphtheriae*; in 1/2 for measles virus; and in 2/5 for hepatitis A. None of the seven children had protective IgG levels against *Bordetella pertussis*.

**Conclusion:**

Immunization during severe congenital proteinuria resulted in variable serological responses, with both vaccine- and patient-related differences. Nephrosis appears not to be a barrier to successful immunization.

## Introduction

Solid organ transplant recipients are prone to infection-related morbidity and mortality and pretransplant vaccinations are therefore widely recommended for this population (1, 2). Although immunization is a feasible way to prevent infections, certain patients are less likely to mount protective immune responses following vaccination.

Congenital nephrotic syndrome (CNS) refers to nephrotic-range proteinuria and edema that manifests within the three first months of life (3). CNS is most commonly caused by genetic defects affecting the glomerular filtration barrier. Mutations of the nephrin-coding gene *NPHS1* are responsible for a particularly severe form of CNS, known as the congenital nephrotic syndrome of the Finnish type (4)*.* Majority of Finnish patients carry homozygous truncating mutations, Fin-major (C.121_122delCT) and Fin-minor (C.3325C>T), in the *NPHS1* gene leading to severe damage in the structures of the nephrin molecule, which is an important part of the podocyte slit diaphragm (5). Such severe forms of CNS are typically resistant to antiproteinuric medication and progress to deterioration of the kidney function within the first years of life (4). The incidence of CNF in Finland is approximately 1 in 8,000 live births, which makes it the commonest reason for a child to undergo kidney transplantation (3, 6). In CNF, an active treatment approach with initial albumin infusions followed by bilateral nephrectomy and early kidney transplantation appears to be the only effective treatment to ensure sufficient growth and development (4).

In CNF, immunizations have traditionally been postponed until bilateral nephrectomy has been performed (7). The rationale for this stems mainly from a hypothetically inferior immunization outcome due to the heavy proteinuria (7).

However, children with severe CNS have an increased risk for infections due to urinary losses of immunoglobulins and other soluble components of the immune system (4). In fact, infections are the primary cause of death in children with CNS (4). Thus, appropriate immunization, especially against encapsulated bacteria, is crucial in children with CNS.

If effective, early prenephrectomy immunization results in protection against vaccine-preventable diseases at an earlier age. Moreover, immunization before nephrectomy allows shorter, minimum 3–4 weeks, dialysis time and possible vaccine-related delays of transplantation are avoided. However, no data exist on the immunological responses of vaccines given during severe congenital nephrosis.

In the present study, we aimed to evaluate vaccine responses in 7 children with CNF with heavy proteinuria who were immunized before nephrectomy. Our hypothesis was that these patients would have detectable antibody levels after nephrectomy as a marker of adequate vaccine response.

## Materials and methods

### Ethics

The study was approved by the scientific committee of the Children's Hospital, Helsinki University Hospital. Register-based studies do not require ethical approval in Finland.

### Patients and data collection

This study was a retrospective descriptive pilot study of seven patients with genetically confirmed CNF who received at least part of their immunizations during nephrosis and were treated at the New Children's Hospital, Helsinki University Hospital.

All patients had a mutation in the *NPHS1* gene and received daily albumin infusions (1–4 g/kg/day), followed by bilateral nephrectomy, dialysis, and kidney transplantation (Table 1). The samples for immune response measurement were collected after nephrectomy. The initial idea of sample collection both before and after kidney transplantation was not possible, because in many cases the total sample volume exceeded 10% of the estimated blood volume (approximately 10 ml), which is the maximum sample volume allowed to draw.

**Table 1A:** Patient characteristics.

Patient no.	*NPHS1* mutation	Age at start of immunization	Age at nephrec-tomy	Age at transplant	Interval from transplant to assessment	Interval from vaccination to assessment	Assessment before (B) or after (A) transplant	HD/PD
1	Maj./Maj.	15	22	22	7	14	A	HD
2	Maj./Mis.	7	20	21	12	23,5	A	HD
3	Maj./Maj.	7	12	13	15	18	A	HD
4	Maj./Maj.	8	25	27	8	22,5	A	PD
5	Maj./Min.	6	14	15	0	3	B	HD
6	Maj./Maj.	7	27	28	0	19	B	HD
7	Maj./Min.	8	16	18	0	6	B	PD

**Table 1B:** Quantitative results of antibody measurements.

Patient no.	Rubella (IU/ml)	C. diphteria (IU/ml)	C. tetani (IU/ml)	Poliov. 1 (titer)	Poliov. 3 (titer)	H.influenzae (µg/ml)	B.pertussis (IU/ml)	HBV^a^ (mIU/ml)	VZV (arb. unit)
1	N/A	0.59	3.2	2,048	1,536	7.7	30	N/A	N/A
2	N/A	0.14	0.13	8	neg	0.28	<40	640	31
3	51	0.04	0.21	neg	neg	0.32	<10	64	neg
4	N/A	0.62	>5.0	380	1,500	17	<40	730	31
5	> 350	0.07	4.8	N/A	N/A	3.3	<40	73	33
6	N/A	0.51	>5.0	770	48	>30.0	<40	17	26
7	N/A	0.01	>5.0	48	12	0.54	<40	N/A	14

Age and interval data are provided in months. Data for interval between last immunization and assessment are presented as median. Solely qualitative results were provided for hepatitis A virus and measles.

arb. unit, arbitrary unit; HBV, hepatitis B virus; HD, hemodialysis; Maj., Fin-major; Min., Fin-minor; Mis, missense; N/A, not available; PD, peritoneal dialysis; VZV, varicella zoster virus; Tx, transplantation.

^a^ Antibody against hepatitis B surface antigen.

In three patients, the serological vaccine responses were assessed before transplantation, whereas in four patients, the responses were measured after transplantation while on immunosuppressive treatment. Patient characteristics are summarized in Table 1. All patients received vaccines according to normal manufacturer provided doses.

### Measurement of immunization response

We evaluated responses to vaccines against the following pathogens: measles, mumps, and rubella (MMR vaccine, *n* = 2); *Corynebacterium diphtheriae, Clostridium tetani, Bordetella pertussis*, poliovirus, and *Haemophilus influenzae* type B (a pentavalent vaccine, *n* = 7); 13 vaccine-type pneumococcal serotypes (PCV13, *n* = 6); Varicella zoster virus (VZV vaccine, *n* = 6); and hepatitis A and B (Twinrix® vaccine, *n* = 5). Serological responses were evaluated only for vaccines delivered during the nephrotic state before nephrectomy.

The analyses were carried out in two accredited clinical laboratories in Finland. Serological analyses were performed by microneutralization assays and enzyme-, luminescence- and fluorescent-microsphere immunoassays. The assays used for each pathogen and the in-house reference values used for estimated protective immunity are presented in Table 2.

**Table 2:** Assays and reference values used for serological assessment.

Pathogen	Method	Cutoff^a^
Measles virus	Immunoluminometric assay (IgG)	pos/neg
Mumps virus	Immunoluminometric assay (IgG)	pos/neg
Rubella virus	Chemiluminescence immunoassay (IgG)	>10 IU/ml
Corynebacterium diphtheriae	Enzyme immunoassay	>0.1 IU/ml
Clostridium tetani	Enzyme immunoassay	>0.1 IU/ml
Polioviruses 1 and 3	Microneutralization assay	<1:8
Haemophilus influenzae (type b)	Fluorescent microsphere immunoassay	>1 ug/ml
Streptococcus pneumoniae^b^	Fluorescent microsphere immunoassay	>0.35 µg/ml
Bordetella pertussis	Enzyme immunoassay (IgG)	>40 IU/ml
Hepatitis B-virus, s-antigen	Immunochemiluminometric assay	>10 mIU/ml
Hepatitis A-virus	Immunochemiluminometric assay	pos/neg
Varicella zoster virus	Enzyme immunoassay	pos/neg

^a^ In-house estimated reference value for protective antibody level.

^b^ Serotypes 1, 4, 5, 6B, 7F, 9V, 14, 18C, 19F, 23F.

The antibody levels were interpreted as sufficient or insufficient for protective immunity according to values presented in Table 2. For a sufficient response to the pneumococcal conjugate vaccine, an antibody level over 0.35 µg/ml was required for at least 9 of the 10 serotypes included in the vaccine.

### Statistical analysis

For the analyses, the vaccine responses were dichotomized into protective vs. non-sufficient. Statistical analyses were performed with SPSS v. 29.0.1.0 (SPSS, Inc., Chicago, IL, USA) applying the Mann-Whitney *U* test for comparisons of continuous variables and the Pearson's chi-square or Fisher's exact test for categorical variables. The level of statistical significance was set at 0.05 for all analyses.

## Results

### Clinical characteristics

Immunizations were started at a median age of 7 months (IQR 7–8), with a concurrent median proteinuria of 36,500 mg/L (IQR 30,900–64,250). Bilateral nephrectomy was performed at a median age of 20 months (IQR 14–25), and the patients underwent kidney transplantation 10–88 days after the nephrectomy. The median interval between immunization and measurement of respective antibody levels was 18 months (IQR 6–23).

### Response to immunization

Protective antibody levels were detected in all examined patients for hepatitis B (5/5), *C. tetani* (7/7), rubella virus (2/2), and mumps virus (1/1); in 5/6 for varicella; in 4/6 for poliovirus and vaccine-type pneumococcal serotypes; in 4/7 for *H. influenzae* type B and *C. diphtheriae*; in 1/2 for measles virus; and in 2/5 for hepatitis A. None of the seven patients had protective IgG levels against *B. pertussis*.

**Figure 1:**
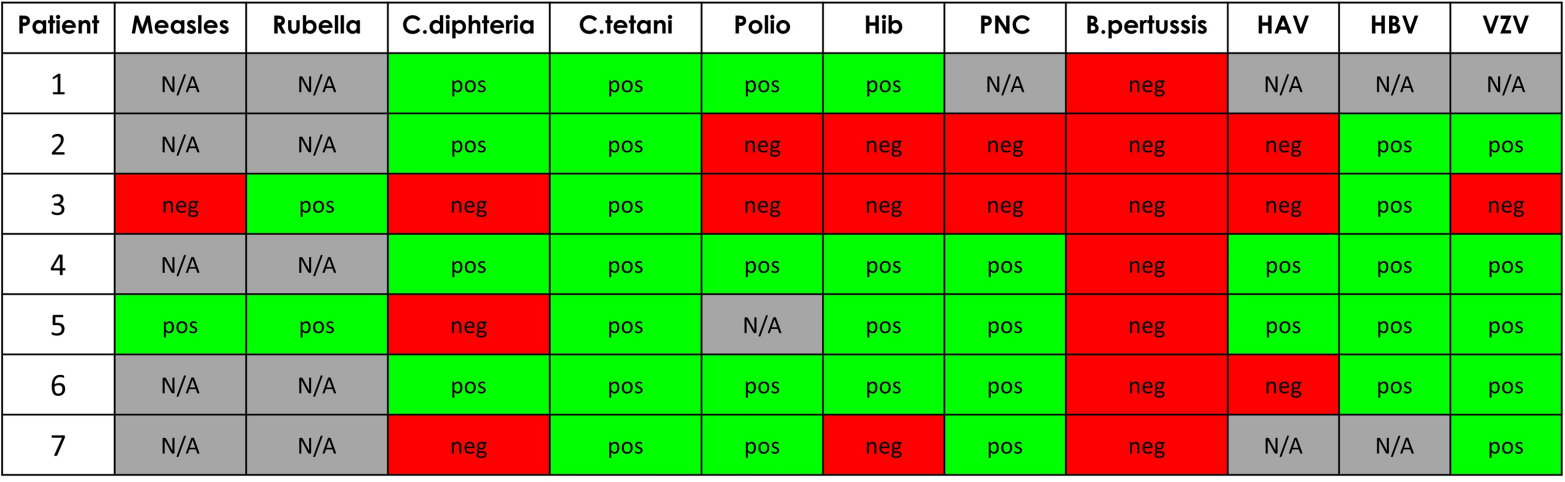
Vaccine responses. HAV, hepatitis A virus; HBV, hepatitis B virus; Hib, *H. influenzae* type B; N/A, not available; PNC, pneumococcus; VZV, varicella zoster virus.

 Figure 1 depicts the serological vaccine responses for each patient and pathogen. Serology for mumps was successfully investigated solely in patient nr. 5, who showed a protective level of IgG antibodies. Quantitative results of the antibody measurements are presented in Tables 1, 3.

**Table 3:** Quantitative pneumococcal vaccine responses.

Serotype	Patient 2	Patient 3	Patient 4	Patient 5	Patient 6	Patient 7
1	0.041	0.05	0.66	1.7	2.4	2
4	0.062	0.12	0.74	1.8	3.5	3.2
5	<0.04	0.067	0.95	1.3	0.83	2.5
6B	0.67	0.22	1.4	0.64	1.9	0.93
7F	0.11	0.45	1.1	4.5	4.1	1.6
9V	0.26	0.11	0.7	1.2	0.21	2.4
14	<0.062	0.076	2.3	5.5	0.7	3.1
18C	0.099	0.15	1.3	1.6	1.9	1.6
19F	0.28	0.22	>30	1.5	3	5.2
23F	0.22	1.1	1.3	0.29	2.1	0.58

Antibody levels are expressed as µg/ml.

Due to the small sample size, we could not perform a multivariate analysis of possible factors influencing the immunization response. However, patients with an inadequate serological response to *C.diphteriae* (patients nr. 3, 5, and 7) were younger both at nephrectomy (median age of 14 vs. 24 months, *p* = 0.03), transplantation (median age of 15 vs. 25 months, *p* = 0.03) and laboratory assessment (median age of 17 vs. 32 months, *p* = 0.05), compared with those having a protective response (Table 1).

While comparing patients whose serological responses were assessed before transplantation (patients nr. 5–7) to those assessed after transplantation (patients nr. 1–4), the former group seemed to elicit a better overall response to immunizations. Excluding *B.pertussis*, the rate of seroprotection was 82.6% (19/23) for patients nr. 5–7 for all examined responses, compared to a corresponding 63.3% (19/30) for patients 1–4 (Figure 1).

### Vaccination related safety aspects

No severe adverse events (SAEs) were notified.

## Discussion

Immunization before kidney transplantation is recommended both in adult and in pediatric transplant recipients (1, 2). However, the current knowledge about vaccine responses in proteinuric children is scarce. The present case series shows for the first time that immunizations given during heavy proteinuria in children with severe CNS resulted in reasonable serological responses, which seemed to reflect both vaccine- and patient-related differences. Nonetheless, seroprotection was reached in at least 40% of patients for the different vaccines excluding pertussis, arguing against nephrosis being a barrier to successful vaccination. By starting immunizations already during nephrosis, we have been able to shorten the dialysis time significantly in children with CNF.

A recent meta-analysis evaluating 90 research articles and case reports including 1,015 patients with nephrotic syndrome concluded that the response to vaccinations was generally good. However, it is of note that not all patients were immunized while being nephrotic (8).

None of our patients had protective levels of IgG antibodies against *B. pertussis*. Previous studies evaluating the pertussis vaccine in nephrotic children are few. Ajay et al. reported a 31.6% seroprotection among 76 children with nephrotic syndrome (9). However, in their study CNS cases were excluded, and the included patients were older than in the present study and had been vaccinated while not nephrotic. Overall, rapid waning of circulating anti-pertussis antibody levels after immunization in small children is a well-recognized problem, and IgG levels against the pertussis toxoid commonly decline to low levels within one year from immunization (10). In our study, the serological response in 5 of the 7 patients was assessed more than 12 months after immunization. Nonetheless, low levels of circulating anti-pertussis antibodies do not necessarily point towards unsuccessful vaccination due to nephrosis or kidney transplantation (11–13).

Poor rates of seroprotection against vaccine-preventable diseases after solid organ transplantation are a well-recognized problem. Urschel et al. showed that protective levels of antibodies against vaccine-preventable diseases were found in 22%–74% of children after heart or heart-lung transplantation, depending on the pathogen (14). Similar results have been obtained after liver transplantation for measles and varicella (15, 16), and after kidney transplantation for *C. diphtheriae*, *C. tetani* and hepatitis B (17). In line with these previous reports, especially patients 2 and 3 in our study showed inadequate responses to several of the examined vaccines, when assessed 12–15 months after transplantation. Although these poor humoral responses against vaccine-preventable diseases after transplantation are partly explained by inadequate pre-transplant immunizations, waning of previously acquired immunity also contributes to these results (2, 17, 18). The precise mechanism for deteriorating humoral immunity after transplantation remains unknown. Nonetheless, follow-up of post-transplant immunization responses seems warranted, and booster doses should be considered on an individual basis (2, 19).

A few details emerge while exploring the differing immunization responses in the studied patients. First, an impact of transplantation and subsequent immunosuppressive treatment on the measured antibody levels seems probable, as patients 5–7 who were assessed before transplantation tended to have a better overall immunization response when compared to patients 1–4 (Table 1, Figure 1). Second, younger age at nephrectomy, transplantation, and laboratory assessment was associated with a poorer serological response to *C.diphteriae* (patients 3, 5, and 7, compared to the others; Table 1).

Factors previously associated with an inadequate immunization response after transplantation include a shorter interval from vaccination to transplantation, younger age at transplantation, and a longer time from transplant to the assessment of the response, among others (14–16, 18). In this aspect, the results of this study are compatible with previous data.

Nephrotic patients have an increased risk for infectious complications such as pneumococcal sepsis (4). Early immunization might provide cell-mediated protection against such infections, despite the loss of immunoglobulins in the urine. 4 of the 6 examined patients in our study showed a good response to the PCV, including all the patients who were assessed before transplantation (Table 3, Figure 1). Due to the post-transplant waning of the humoral response, we nowadays give a booster dose of PCV13 to all patients 6 months after the transplantation (20).

An interesting finding of this preliminary study was clearly detected response to Varicella vaccination (5/6) without any immunization related adverse effect even despite gross proteinuria. Live vaccines are suggested to be given minimum 4 weeks before transplantation, which in our protocol would be simultaneously with nephrectomy. Based on our cases series, we continue to give live vaccines during nephrosis well before major surgery such as nephrectomy or transplantation.

The safety and effectiveness of vaccines given during nephrotic range proteinuria have raised concern among clinicians. In our patients, no SAEs were reported. The meta-analysis by Angeletti et al. supports this finding by concluding that all types of vaccines can be safely used in children with acute onset nephrotic syndrome (8).

Our study comes with some limitations. The small sample size and non-complete data for some vaccines limits further analyses of the differences noted between both children and the measured responses. While 4 of the 7 patients were assessed after transplantation, post-transplant waning of the humoral response cannot be distinguished from unsuccessful primary immunization in these patients. Finally, as we were unable to investigate cell-mediated immune responses to immunizations, our results can only be interpreted concerning the humoral part of the response.

## Conclusion

Our data suggest that immunizations given during severe congenital nephrosis result in variable serological responses, reflecting both vaccine- and patient-related differences. Indeed, despite losing the newly formed antibodies in the urine while nephrotic, these children seem to mount a sustained immunological response with circulating protective levels of antibodies against most of the pathogens once the proteinuria has resolved. However, follow-up of the serological responses seems warranted. Immunization during severe congenital nephrosis thus seems to present a viable option when in need, although larger studies are needed to confirm the results and a long-term immune response.

## Data Availability

The datasets presented in this article are not readily available due to privacy protection issues. Requests to access the datasets should be directed to Okko Savonius, okko.savonius@helsinki.fi.
